# High-Quality Draft Genome Sequence of Xanthomonas arboricola pv. juglandis CPBF 1521, Isolated from Leaves of a Symptomatic Walnut Tree in Portugal without a Past of Phytosanitary Treatment

**DOI:** 10.1128/MRA.00887-18

**Published:** 2018-10-25

**Authors:** Camila Fernandes, Jochen Blom, Joël F. Pothier, Fernando Tavares

**Affiliations:** a CIBIO, Centro de Investigação em Biodiversidade e Recursos Genéticos, InBIO Laboratório Associado, Microbial Diversity and Evolution Group, Universidade do Porto, Vairão, Portugal; b Unidade Estratégica de Investigação e Serviços de Sistemas Agrários e Florestais e Sanidade Vegetal, Instituto Nacional de Investigação Agrária e Veterinária, Oeiras, Portugal; c Faculdade de Ciências, Departmento de Biologia, Universidade do Porto, Porto, Portugal; d Bioinformatics and Systems Biology, Justus-Liebig-University Giessen, Giessen, Germany; e Environmental Genomics and Systems Biology Research Group, Institute for Natural Resource Sciences, Zurich University of Applied Sciences (ZHAW), Wädenswil, Switzerland; University of California, Riverside

## Abstract

Here, we report the draft genome sequence of Xanthomonas arboricola pv. juglandis CPBF 1521, isolated from symptomatic leaves of an ornamental walnut in a public site in Portugal without any record of phytosanitary treatment. This isolate may constitute a genomic reference of a wild-type strain in comparative genomics studies.

## Abstract (precis)

Here, we report the draft genome sequence of Xanthomonas arboricola pv. juglandis CPBF 1521, isolated from symptomatic leaves of an ornamental walnut in a public site in Portugal without any record of phytosanitary treatment.

## ANNOUNCEMENT

Xanthomonas arboricola pv. juglandis (Gammaproteobacteria class, Xanthomonadales order, Xanthomonadaceae family) is a threatening and important pathogen of the principal commercial nut trees Persian walnut and English walnut (Juglans regia L.) (1, 2). Diseases caused by X. arboricola pv. juglandis have been demonstrated by the development of several symptoms, namely the presence of necrotic lesions on leaves and fruits, the presence of external apical necrosis near the blossom end evolving into fruit necrosis, and the presence of vertical cankers, brown to black exudates, and distortions on trunks (3–5). Not surprisingly, X. arboricola pv. juglandis is responsible for increasing losses in walnut production resulting in a negative economic impact for walnut crop regions in many countries worldwide (1, 2, 6).

The present announcement reports the whole-genome sequence of a X. arboricola pv. juglandis strain, CPBF 1521, isolated in October 2014 from the leaves of an ornamental J. regia specimen in a public site in Loures, Portugal, showing typical symptoms of walnut bacterial blight, and for which no phytosanitary treatments were applied. This set of features suggests that this strain has not been exposed to selective pressures caused by phytosanitary treatments, such as copper-based compound sprays, making this genomic data set particularly interesting for comparative genomics studies.

X. arboricola pv. juglandis CPBF 1521 was obtained from infected leaf samples as previously described (7) and was grown on M2 medium (yeast extract, 2 g liter^−1^; Bacto peptone, 5 g liter^−1^; NaCl, 5 g liter^−1^; KH_2_PO_4_, 0.45 g liter^−1^; and Na_2_HPO_4_ 12H_2_O, 2.39 g liter^−1^) at 28°C for 48 h with shaking (100 rpm). The EZNA bacterial DNA purification kit (Omega Bio-Tek, Norcross, GA) was used for DNA extraction. Standard genomic library preparation and sequencing was carried out with at the GATC Biotech AG (Konstanz, Germany) using an Illumina HiSeq platform with 2 × 150-bp paired-end reads. Raw sequence data with approximately 10,113,730 reads were assembled *de novo* using MIRA version 4.0 (8) in accurate mode with standard settings. The set of contigs obtained was reassembled using SeqMan Pro from the Lasergene genomics package version 12.1.0 (DNAStar, Madison, WI) with Pro Assembler parameters and reads were mapped using SeqMan NGen with standard settings to check for inconsistencies. Contigs were ordered according to the related reference genome of X. arboricola pv. juglandis (strain CFBP 2528; GenBank accession number NZ_JZEF00000000) (9) using the Move Contigs function in Mauve version 20150226 build 10 (10, 11). Genome annotation was performed with Prokka version 1.12 (12) based on *de novo* discovery of genes using a Xanthomonas genus database.

The X. arboricola pv. juglandis CPBF 1521 genome contains 56 contigs with an *N*_50_ value of 173,159 bp, has a total size of 5,194,740 bp, and a G+C content of 65.41%. A total of 4,494 coding sequences (CDS) with 4 rRNAs and 53 tRNAs were found using Prokka. Initial comparative analysis using EDGAR version 2.0 (13) identified 3,984 CDS shared between CPBF 1521 and X. arboricola pv. juglandis CFBP 2528 that was used as a reference genome. Moreover, a high average nucleotide identity value (98%) was obtained between these two strains.

This newly sequenced genome expands the existing genomic data for X. arboricola pv. juglandis (8, 14–16). Further genomic comparison studies are being conducted to identify unique genomic features of X. arboricola pv. juglandis CPBF 1521.

### Data availability.

This whole-genome shotgun project has been deposited in the European Nucleotide Archive (ENA) under the BioProject accession number PRJEB27248 and in GenBank under the accession number UIHD00000000. The version described in this paper is the first version, UIHD01000000.
